# NaviCell Web Service for network-based data visualization

**DOI:** 10.1093/nar/gkv450

**Published:** 2015-05-09

**Authors:** Eric Bonnet, Eric Viara, Inna Kuperstein, Laurence Calzone, David P. A. Cohen, Emmanuel Barillot, Andrei Zinovyev

**Affiliations:** 1 Institut Curie, 26 rue d'Ulm, 75248 Paris, France; 2 INSERM, U900, 75248 Paris, France; 3 Mines ParisTech, 77300 Fontainebleau, France; 4 Sysra, 91330 Yerres, France

## Abstract

Data visualization is an essential element of biological research, required for obtaining insights and formulating new hypotheses on mechanisms of health and disease. NaviCell Web Service is a tool for network-based visualization of ‘omics’ data which implements several data visual representation methods and utilities for combining them together. NaviCell Web Service uses Google Maps and semantic zooming to browse large biological network maps, represented in various formats, together with different types of the molecular data mapped on top of them. For achieving this, the tool provides standard heatmaps, barplots and glyphs as well as the novel map staining technique for grasping large-scale trends in numerical values (such as whole transcriptome) projected onto a pathway map. The web service provides a server mode, which allows automating visualization tasks and retrieving data from maps via RESTful (standard HTTP) calls. Bindings to different programming languages are provided (Python and R). We illustrate the purpose of the tool with several case studies using pathway maps created by different research groups, in which data visualization provides new insights into molecular mechanisms involved in systemic diseases such as cancer and neurodegenerative diseases.

## INTRODUCTION

Biology is a scientific discipline deeply grounded in visual representations serving for communicating results and ideas. Nowadays, there is a strong incentive to provide *interactive* web-based visual representations, that users can easily adapt to address a biological question. Modern molecular biology is particularly demanding for new tools that can represent numerous ‘omics’ data in a meaningful way. Visualizing quantitative ‘omics’ data in the context of biological networks provides insights into the molecular mechanisms in healthy tissues and in diseases (1,2). This is one of the most demanded features of existing pathway databases such as KEGG, Reactome and BioCyc (3–5). To answer this need, many tools have been developed allowing mapping ‘omics’ data on top of biological networks (6–9). These tools use the content of existing pathway databases to retrieve the pathway information through dedicated application programming interface (API). In addition, some pathway databases provide graphical user interfaces (GUI) to perform data visualization, on interactive pathway maps.

When a user faces the necessity to visualize the ‘omics’ data on biological networks, there are currently only two options available. The first is to use a graphical interface of a suitable pathway database, which can be a tedious manual work. The second is to use one of the available standalone or web-based tools for creating static images of colored pathways which are not interactive anymore, and do not allow browsing the molecular interactions. Currently, there is a lack of well-developed APIs that allow applying data visualization programmatically on top of biological networks, such that the ‘omics’ data can be browsed simultaneously with the pathway information.

Available network-based methods for data visualization have several common limitations. First, most of them do not provide any possibility of data abstraction and do not provide a possibility of visualizing coarse-grained trends in the ‘omics’ data. This is needed if, for example, there is a wish to visualize the whole transcriptome of a cell or a group of samples on a large network, representing an important part of the cellular interactome. Current methods usually attach some elements of the standard scientific graphics (gradient color, heatmaps, barplots) to the individual elements of pathway maps, which makes the visualization hardly readable at higher levels of zooming as well as the map content itself. Second, most of data visualization tools are specific to a particular pathway database structure, format and graphical pathway representation. This limits the use of data visualization on user-defined maps. Third, APIs for programmatic web-based data visualization are rudimentary or do not exist.

To overcome some of these limitations, we have developed NaviCell Web Service tool. It allows visualizing ‘omics’ data via GUI and flexible API using standard and advanced methods of data visualization, including a possibility to perceive the mapping of data at different network scales. The NaviCell Web Service can exploit pathway maps created by users or from existing databases, including large network maps with thousands of elements.

The NaviCell Web Service for Network-based Data Visualization is a combination of (i) a user-friendly NaviCell JavaScript-based web interface (10), allowing browsing large maps of biological networks using Google Maps API and *semantic zooming* principle, and visualizing ‘omics’ data on top of them (https://navicell.curie.fr); (ii) a collection of maps available for data visualization including the Atlas of Cancer Signaling Network (ACSN) (https://acsn.curie.fr) and other detailed network maps created by research groups for particular topics (Toll-like receptor signalling, EGFR and mTOR pathways, mast cell activation, dendritic cells, iron metabolism, Alzheimer disease and others); (iii) a REST API allowing programmatic use of all data visualization functions and manipulating the web interface.

A quick ‘live example’ which *automatically* uploads a sample dataset from TCGA database and illustrates the data visualization capabilities of NaviCell Web Service is provided at http://navicell.curie.fr/pages/nav_web_service.html. Sample Python code for quick start on using NaviCell Web Service API is available from the same link in the ‘Python API and data files’ section. An example screenshot of NaviCell Web Service GUI can be found in the manual located at the same link.

NaviCell Web Service code and APIs are distributed under LGPL licence.

## ALGORITHMS AND SOFTWARE

### Implementation

We conceived the NaviCell Web Service for Network-based Data Visualization as a significant development of NaviCell network browser (10). New functionalities include possibility of visualizing and analyzing different types of ‘omics’ data, from within NaviCell interface, and a set of programmatic APIs allowing to manipulate all NaviCell features.

**Figure 1.**
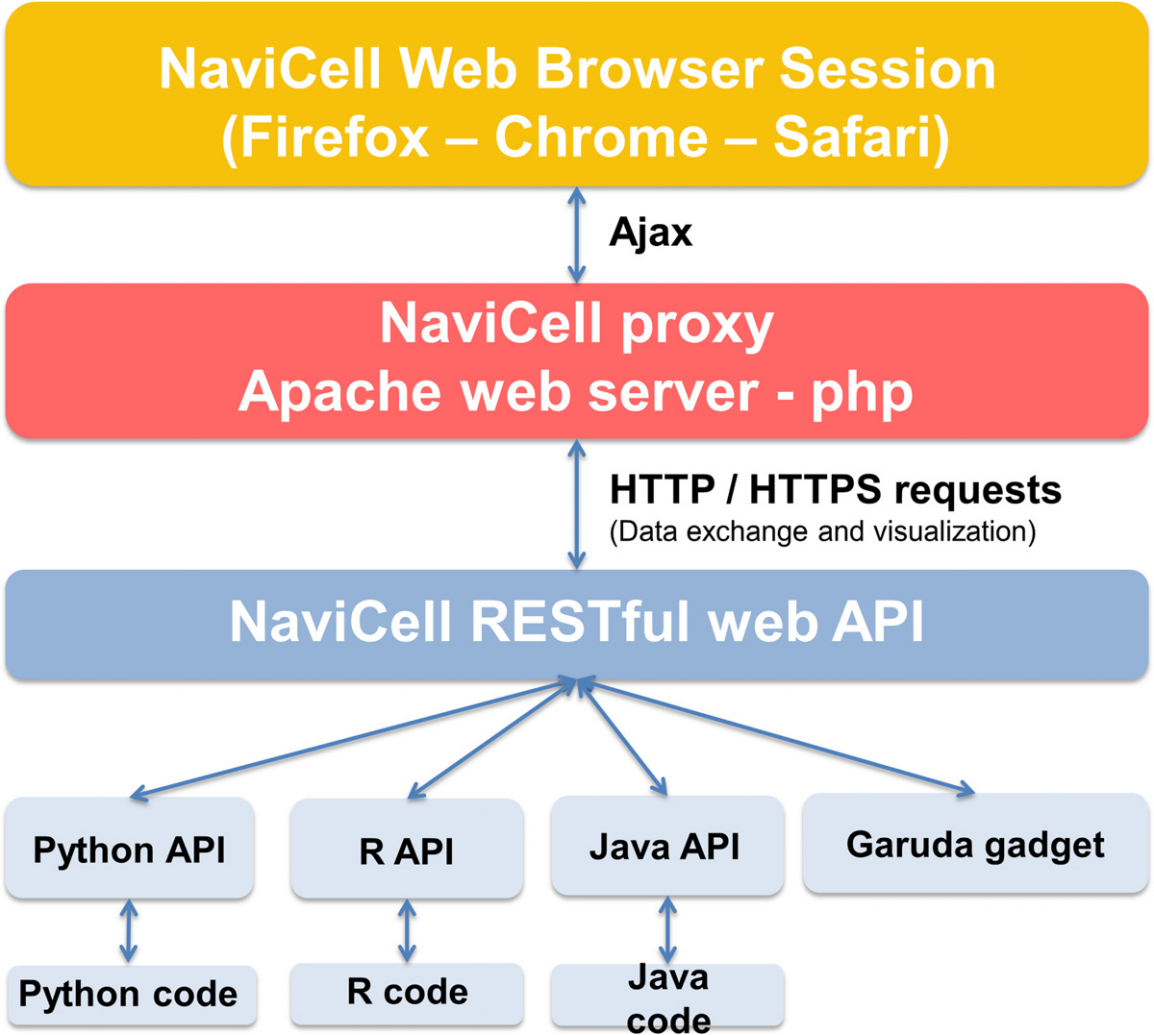
General architecture of the NaviCell Web service *server*. Client software (light blue layer) communicates with the server (red layer) through standard HTTP requests using the standard JSON format to encode data (RESTful web service, dark blue layer). A session (with a unique ID) is established between the server and the browser (yellow layer) through Ajax communication channel to visualize the results of the commands send by the software client. It is worth noticing that communication channels are bidirectional, i.e. the client software can send data (e.g. an expression data matrix) to the server, but it can also receive data from the server (e.g. a list of HUGO gene symbols contained in a map).

The web service is implemented at two interconnected functional levels. The first level corresponds to the interactive, dialog-based use of the web interface to load and visualize data. This level is implemented as a specific set of JavaScript functions linked to the menu located in the right-hand panel of the NaviCell web interface. The second level corresponds to a programmatic usage of the service, which allows users to write the code that will communicate with the NaviCell *server* in order to automate all the visualization operations. This functional level is implemented as a RESTful web service, using standard web protocol HTTP operations and data encoding (JSON) to perform all the necessary operations (11). RESTful web services are very popular having the advantage of being lightweight, efficient and simple be use and to be implemented in different programming languages. The API for NaviCell Web Service is implemented in Python and R, and Java API is in development. These APIs will facilitate the integration of NaviCell Web Service in other applications: among them, there is ongoing work on providing the data visualization services through the Garuda Alliance web platform, which aims to be a one-stop service for bioinformatics and systems biology (12) and GeneSpring software (13). Figure 1 summarizes the NaviCell Web Service architecture and information flow.

### Input data types

The pathway maps which can be used for NaviCell Web Service data visualization can be prepared using CellDesigner tool (14), which is widely used in systems biology community and based on the Systems Biology Graphical Notation standard (15) for representing biological networks. These pathway maps are converted into interactive Google Maps-based web-interfaces via NaviCell tool (10). Other pathway map formats can be used in NaviCell through dedicated converters implemented in BiNoM Cytoscape plugin (16,17). They extend the functionalities of NaviCell to practically any network that can be imported into Cytoscape environment, though careful and sometimes time-consuming design of the network layout is required in order to obtain meaningful and interpretable network support for data visualization.

NaviCell data visualization web service is able to process several types of ‘omics’ data, see Table 1. The different biological data types are mapped into several internal representations that determine what methods of data visualization can be applied.

**Table 1.** List of biological input data types accepted by NaviCell Web Service

‘Omics’ data type	Internal representation
mRNA expression data	Continuous
microRNA expression data	Continuous
Protein expression data	Continuous
Discrete copy-number data	Discrete ordered
Continuous copy-number data	Continuous
Mutation data	Discrete unordered
Gene list	Set

The first column lists the types as they appear in the interface. The second column lists the internal data representations that are used to determine what type of data visualization can be applied.

For instance, a mRNA expression data matrix is associated to a ‘continuous’ numerical internal representation. Thus, if the user chooses to display this data with a heatmap, mapping to a color gradient will be applied by default, with a possibility to modify the default settings. On the other hand, when a matrix with discrete copy-number data is loaded, it is associated with a ‘discrete ordered’ internal representation for which a specific color palette is applied for visualization, with a distinct color associated to each discrete copy-number state.

The input format for data sets is standard tab-delimited text files, with rows representing genes, their products or metabolites and columns representing samples (or experiments, or time points). Genes in the first column should be labeled by their standard HUGO (HGNC) gene symbols, that will be associated to the different entities on the pathway map.

Users can also upload sample annotations (simple tab-delimited text files) to specify how samples can be organized into meaningful groups (e.g. disease versus control). An appropriate method will be used to summarize the values of all the samples contained in a group, defined by the internal data representation. For instance, expression values for a group of samples are averaged by default for the ‘continuous’ data type. By contrast, for mutation data (‘discrete unordered’ internal data type), ‘at least one element of the group is mutated’ grouping method will be suggested.

### Graphical data representations

We have included in the NaviCell Web Service several methods for graphical representation of molecular data, both broadly used in molecular biology (heatmaps and barplots) and original ones such as *map staining* that have not been previously employed in the context of network-based data visualization.

**Figure 2.**
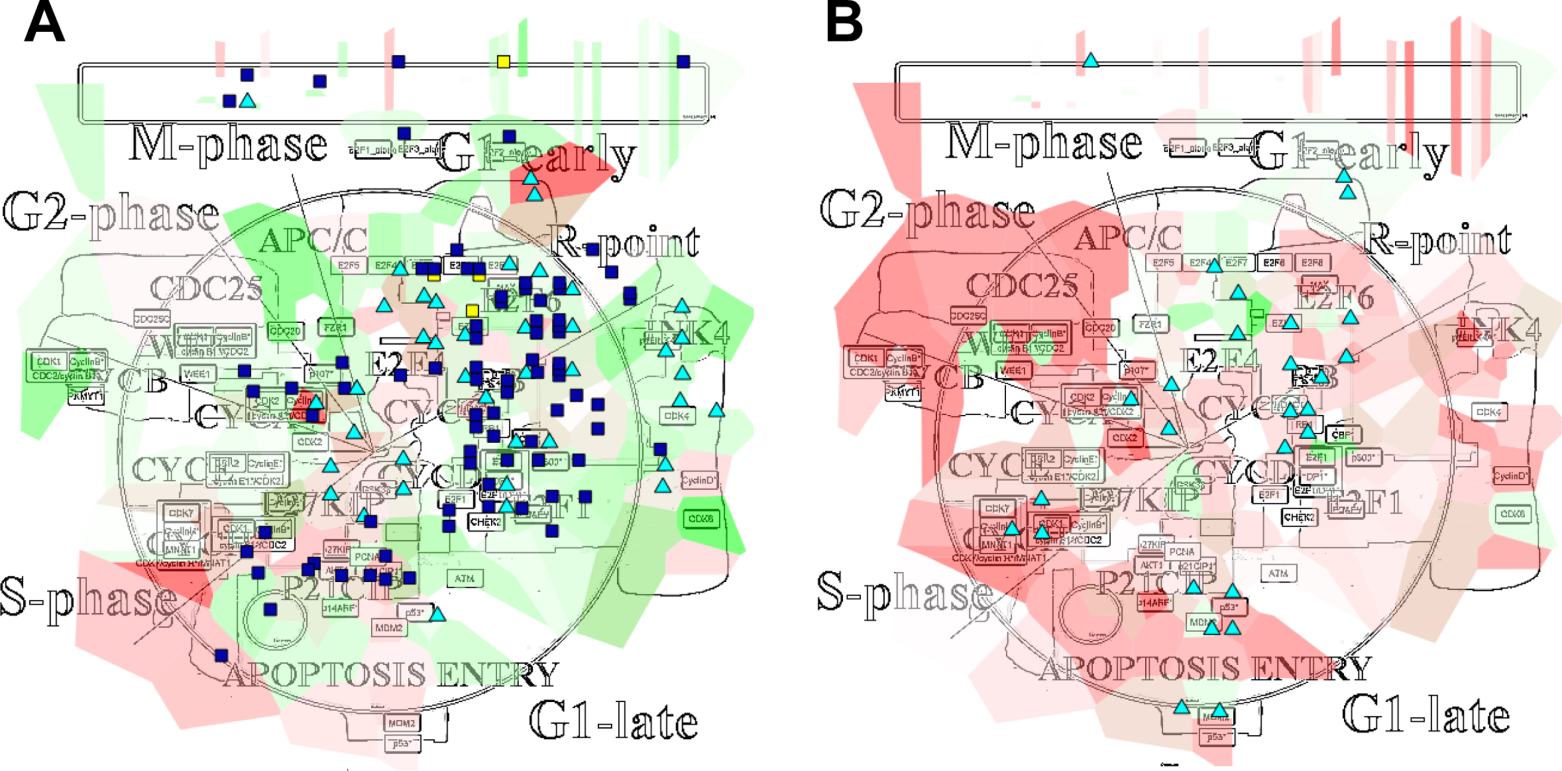
Visualization of multiple data types for two different prostate cancer cell lines. Transcriptomic, gene copy-number values and gene mutations (24) are mapped on the Cell Cycle map. (**A**) Hormone-sensitive prostate cancer cell line (LNCaP). (**B**) Hormone-refractory prostate cancer cell line (DU145). Expression data is visualized using the map staining technique, i.e. colored territories around entities, ranging from low (green) to high expression values (red). Copy-number values are represented by glyphs (squares) with blue color indicating gene loss (values of −1 and lower) and yellow color indicating amplification (values of 1 and higher). Mutated genes are depicted by cyan triangles.

**Figure 3.**
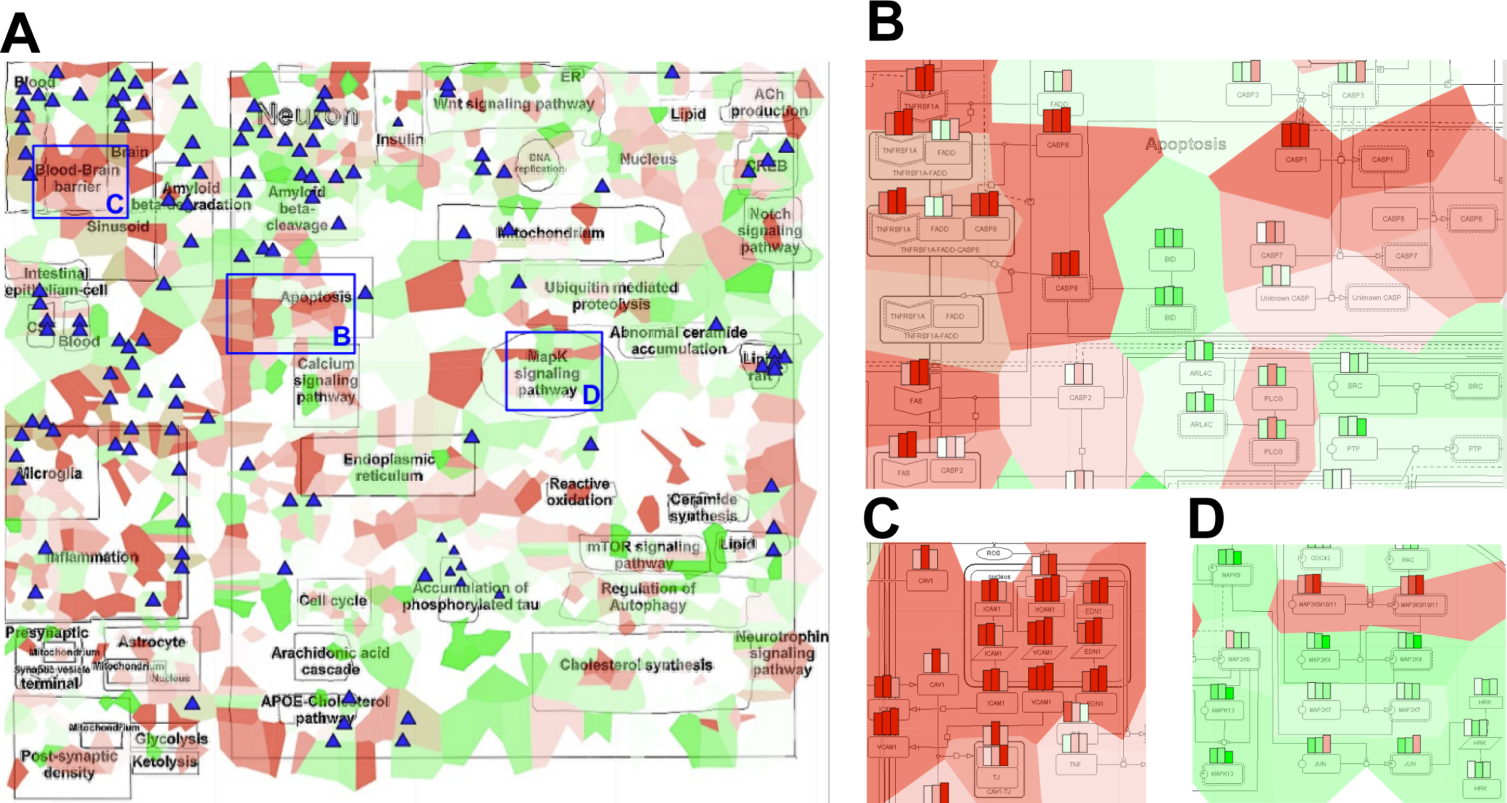
Visualization of two different data types on the Alzheimer's disease (AD) pathway map. (**A**) Top-level view expression data are visualized with map staining (see Figure 2 legend). Frequently mutated genes are indicated by glyphs (blue triangles), with the size of the glyph proportional to the mutation frequency. (**B–D**) represent zooms on known key regulators of (B) apoptosis, (C) blood brain barrier and (D) MAPK signaling pathway. Background colors represent expression values as in (A), while barplots illustrate tissue-specific expression values for frontal cortex (FC), thalamo-cortical area (TC) and hippocampus (HI).

- **Simple markers** are pictograms (similar to the ones used in Google Maps to indicate the geographical locations) drawn on the pathway map at the location of a given molecular species (protein, gene, complex, phenotype). They are used to display the results of a search performed by the user or to display a list of gene names mapped onto the biological network (including species that are part of a complex, or different forms of a given protein).
- **Heatmaps** are often used in molecular biology, in particular for displaying expression data by color gradients. In NaviCell Web Service, heatmaps can be used to visualize continuous data types such as expression values, or discrete data such as copy-number or mutation values. The user can arrange the visualization to show several samples or groups of samples from several data sets (for example, for showing simultaneously gene promoter methylation and expression values).
- **Barplots** are charts with rectangular bars whose height is proportional to the values they represent. Barplots can be efficient to visually distinguish numerical values between two or more conditions (e.g. disease versus control as in Figure 3B–D).
- **Glyphs** are graphical representations using basic geometrical shapes (triangle, square, circle, etc.) and their size and color to visually combine different types of data. For example, the shape of the glyphs can be assigned to the copy-number values, while the expression data can be used to define the color of the glyph. Up to five glyphs can be attached to the same molecular entity, which can be useful to compare several experimental conditions.
- **Map staining** is a novel network-based data visualization method where background areas (or territories) around each molecular entity are colored according to the data value associated to this entity. The area occupied by a particular map element is defined as the Voronoi's cell associated to this element. The Voronoi's cell is a convex polygon containing all the points of the territory which are closer to the chosen element than to any other element (18). In our case, we use all the entities present on the pathway map for defining Voronoi's cells. The polygons are pre-computed for each map and post-processed to avoid too large polygons. They are colored according to the data values and to the options defined by the user. The colorful decorations of the pathway map are replaced by a black-and-white background in order to avoid mixing the colors. Using map staining allows visualizing the large-scale trends in molecular data mapped on top of the biological networks (see examples in Figures 2 and 3).

### Performing statistical analyses from within NaviCell Web Service GUI

Data visualization using biological networks is a powerful method to gain insights into functioning of biological mechanisms. However, the intrinsic capability of human brain to detect complex patterns in distributions of colors, points, etc., can lead to overfitting and poorly supported conclusions. This is to say that all hypotheses formulated from visual data inspection must be supported by rigorous statistical analysis. We provide two examples of such follow-up analyses in Supplementary Materials.

In order to quickly verify ‘impressions’ from data visualization, NaviCell GUI provides a possibility of simple statistical analysis of the uploaded data (‘Functional analysis’ button). For example, the standard hypergeometric test applied to any uploaded gene list allows computing the enrichment *P*-values for all modules of the map, and, therefore, selecting the most interesting module or map for further data visualization and analysis. The list of available data analysis procedures will be continuously extended in the future.

## RESULTS

We show that NaviCell Web Service can be used for visualization of different types of high-throughput data in two case studies:

- comparing two prostate cancer cell lines using the cell cycle map, focusing on transcriptomics and mutation data.
- using the map of molecular interactions involved in Alzheimer's disease (AD) (19) for visualizing the transcriptome data collected for different brain areas (20).

Furthermore, several examples of using NaviCell Web Service for data visualization can be found in NaviCell Web Service user guide and the case studies provided at http://navicell.curie.fr/pages/nav_web_service.html. In Supplementary Materials, we provide two case studies demonstrating visualization of ovary cancer data obtained from The Cancer Genome Atlas (21) on the large map of Atlas of Cancer Signalling Network (22) and an example of using the non-CellDesigner network map of the Ewing's sarcoma signalling network (23) for visualizing transcriptomic time series data.

### Comparing two prostate cancer cell lines

As an illustration of the use of data visualization on top of the network maps for comparing two cancer genomic profiles, we selected two prostate cancer cell lines from the Cancer Cell Line Encyclopedia (24): a prostate hormone-sensitive tumor cell line (LNCaP) and a prostate hormone-refractory tumor cell line (DU145). We gathered gene expression, copy-number and mutation data for these two cell lines and mapped them onto the cell cycle map (25).

Application of map staining data visualization technique allows concluding that most LNCaP cells express genes from the early G1 phase (Figure 2A, upper-right area) and the G1-S checkpoint (Figure 2A, lower-left area), while most DU145 cells express genes from the later stages of the cell cycle (Figure 2B, G1-late, S-phase and G2-phase areas). Mutation visualization shows that few cell cycle genes are mutated, gained, amplified or lost in these two cell lines (Figure 2A and B, square and triangle glyphs), especially in DU145. By zooming in, we can determine that for the LNCaP cells, the most important gene alterations are the amplification of DP1 (present in most complexes involving E2F1), and the homozygous loss of E2F2 transcription factor. The mutations concern genes from the apoptotic pathway: ATR or CHEK2. As for the expression, the more noticeable trend is that the expression of the cell cycle inhibitors, such as Rbl2, p21, Cdc25c, is still high, whereas the expression of the G2 cyclins, for instance CyclinA, is low.

For the DU145 cells, genes involved in later stages of the cell cycle are more expressed compared to the LNCaP cells. It can be that if cells were arrested in LNCaP, they are more advanced in the cycle in DU145 cells by overpassing the G1/S checkpoint and are arrested at the spindle checkpoint. They are more prone to proliferation than the LNCaP cells. The expression of some cyclins seems to confirm this fact: CyclinB, CyclinD and CyclinH are higher than in LNCaP. Some means to stop the cycle seem to be kept though, with high expression of Cdc20 and Cdc25.

### Visualization of transcriptomes of brain cortex regions in AD

We use the AD network map (19) to visualize the expression data from AD patients (20) and mutation data of most frequently mutated genes in AD (taken from http://www.alzforum.org, http://www.molgen.ua.ac.be) (Figure 3). The AD/control expression ratio from Thalamocortical area (TC) is displayed using map staining, whereas glyphs identify mutated genes. The top-level view of the map (where details of the interactions are hidden) shows patterns of expression and illustrates how mutated genes are distributed across different molecular mechanisms. For a more detailed description of the processes, we use bar plot display mode to compare gene expression in samples from Frontal cortex (FC), TC area and Hippocampus (HI) areas (Figures 3B and D). It confirms elevated apoptosis (caspase cascade expression is high, Figure 3B) and downregulated MAPK pathway (Figure 3D). In addition, AD samples are enriched with vascular inflammatory genes ICAM1, VCAM1, EDN1 and TNF, which evidences the Blood–brain barrier breakdown and inflammatory immune response activation in the brain tissue (Figure 3C).

## DISCUSSION AND CONCLUSION

NaviCell Web Service shall contribute to the growing set of highly demanded tools for molecular biology allowing visualization of ‘omics’ data in the context of biological network maps.

We have compared the features of the NaviCell Web Service for data visualization with the web-based tools providing similar functionalities (3–5,26–27), i.e. easy pathway browsing functions and interactive data visualization capabilities. We have focused on the features related to advanced map navigation, molecular data types, graphical representations and programmatic access. The results of this comparison show that the NaviCell Web Service's strengths are: multiple possibilities for graphical representations, support of multiple data types and extended capabilities for programmatic access from different computer languages (Table 2).

**Table 2.** Comparison of the NaviCell Web Service features with similar web sites for pathway-based data visualization

Features	Na	Re	KE	iP	Bc	Pa
Map: advanced navigation	•	•		•	•	•
Map: simple zooming	•	•	•	•	•	•
Map: semantic zooming	•					
Visualization: node coloring		•				•
Visualization: heatmaps	•				•	
Visualization: barplots	•				•	
Visualization: glyphs	•					
Visualization: map staining	•					
Data mapping: gene lists	•	•	•	•	•	•
Data mapping: expression data	•	•			•	•
Data mapping: copy-number data	•					
Data mapping: mutation data	•					
Data mapping: metabolomic data		•			•	
Data mapping: interactions		•				
Programmatic access: RESTful web	•	•	•		•	
Programmatic access: data visual.	•				•	

Abbreviations: Na: NaviCell Web Service, Re: Reactome (4), KE: KEGG (3), iP: iPath (26), Bc: BioCyc (5), Pa: PATIKAweb (27).

## SUPPLEMENTARY DATA

Supplementary Data are available at NAR Online.

SUPPLEMENTARY DATA
