# Supplementary material for: NaviCell Web Service for network-based data visualization
Source: Nucleic Acids Res. 2015 May 9;43(Web Server issue):W560–5. doi: 10.1093/nar/gkv450 (PMC4489283; doi:10.1093/nar/gkv450)
Supplement: SUPPLEMENTARY DATA [file supp_43_W1_W560__index.html]

NaviCell Web Service for network-based data visualization — SUPPLEMENTARY DATA 

# NaviCell Web Service for network-based data visualization

## SUPPLEMENTARY DATA

- SUPPLEMENTARY DATA
