# Supplementary material for: NaviCell Web Service for network-based data visualization
Source: Nucleic Acids Res. 2015 May 9;43(Web Server issue):W560–5. doi: 10.1093/nar/gkv450 (PMC4489283; doi:10.1093/nar/gkv450)
Supplement: SUPPLEMENTARY DATA [file supp_gkv450_nar-00385-web-b-2015-File010.pdf]

Supplementary Materials for the paper

NaviCell Web Service for Network-based Data Visualization

Eric Bonnet [1,2,3], Eric Viara [4], Inna Kuperstein [1,2,3] , Laurence Calzone [1,2,3] , David PA Cohen [1,2,3] , Emmanuel Barillot [1,2,3], Andrei Zinovyev [1,2,3]*

[1]Institut Curie, 26 rue d'Ulm, 75248 Paris, France, [2]INSERM U900, 75248 Paris, France, [3]Mines ParisTech, 77300 Fontainebleau, France, [4]Sysra, 91330 Yerres, France.

CASE STUDY 1: Using NaviCell Web Service for visualization of "omics" data from four intrinsic types of ovarian cancer in the context of ACSN maps

This case study demonstrates the features of NaviCell Web Service applying for visualization of data on very big maps of molecular interactions

Ovarian cancer is the most lethal gynecologic tumor characterized by high heterogeneity in term of genetic background, morphological features and molecular mechanisms involved in the disease initiation and progression. With attempt to classify ovarian cancer, the Cancer Genome Atlas (TCGA) study on integrated genomic analysis of 489 cases has been performed. It revealed four robust signatures that together with patient clinical data and morphological characteristics of tumors gave rise to four intrinsic ovarian cancer types including differentiated, immunoreactive, mesenchymal and proliferative.

To clarify molecular mechanisms standing behind the genetic signatures in the four ovarian cancer types, we have integrated "omics" data from the aforementioned study, the transcriptome, copy number and mutation profiles, into the Atlas of Cancer Signalling Network (ACSN) using the NaviCell Web service visualization techniques.

The expression data visualized as map stainings shows clear differences in the pattern of biological processes regulation across four ovarian cancer types (Figure 1.1). The differentiated ad mesenchymal types are characterized by low proliferative activity, whereas immunoreactive and especially proliferative types exhibit high cell cycle activity that is also associated with increased expression of DNA repair-related genes as positive loop response to accumulated genomic instability in highly cycling cells.

Mesenchymal, the most invasive among the four ovarian cancer types, shows high expression activity in the signaling related to the epithelial to mesenchymal transition (EMT) and cell motility. The proliferative type is rather characterized by elevated expression in the survival pathways (Figures 1.1 and 1.2).

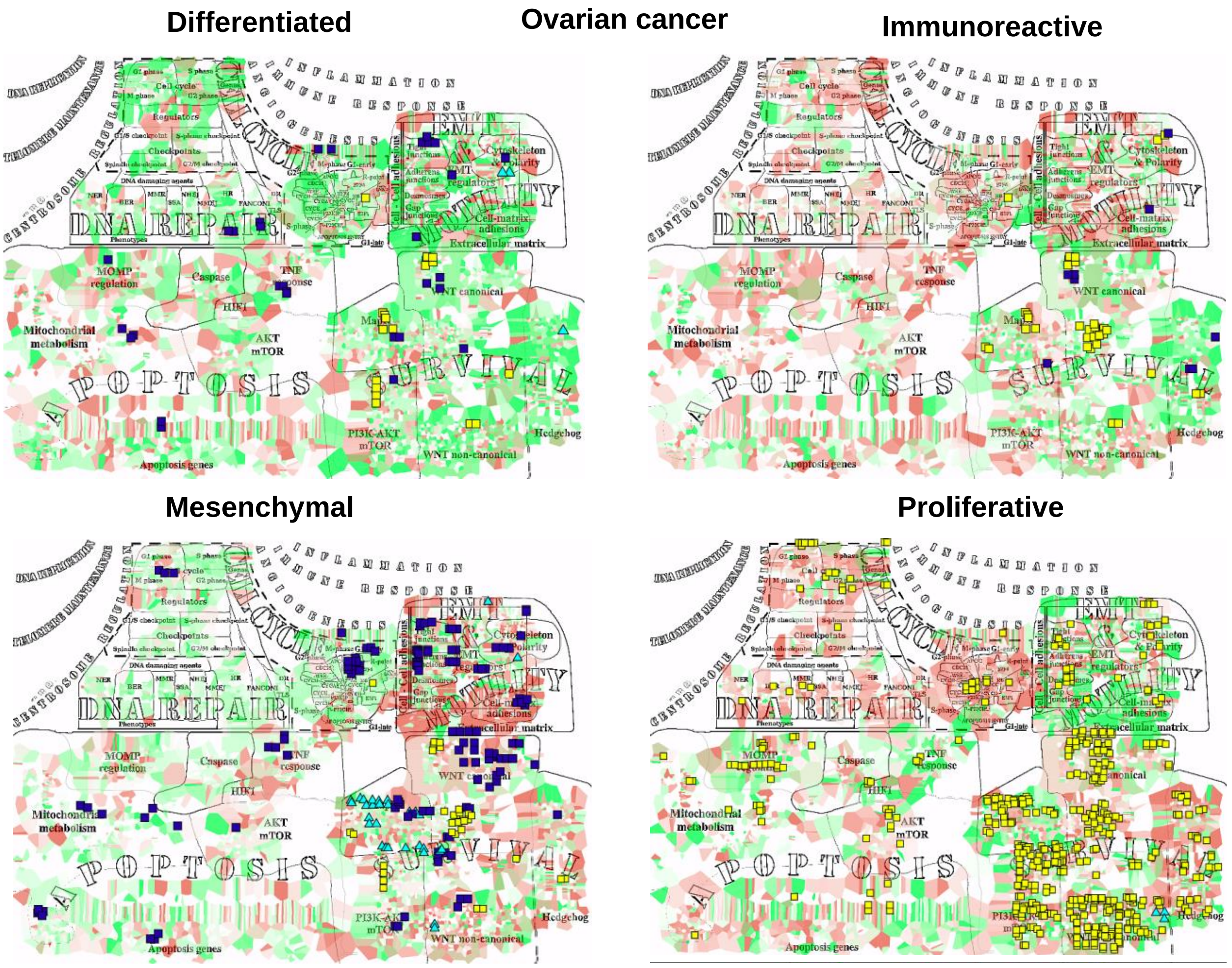

Figure 1.1. Simultaneous visualization of three types of data for four intrinsic types of ovarian cancer. mRNA expression is demonstrated as map staining. Copy number variations are represented as heat maps where yellow squares indicated copy number gains and dark blue indicate copy number losses. Mutations are represented using glyphs (cyan triangles). The data is displayed at the top level zoom demonstrating a pattern of data distribution across biological processes.

Distribution of copy number variations is also not equal across the four types of ovarian cancer (Figures 1.2 and 1.3). For example, mesenchymal type is characterized by multiple copy number losses in the genes related to cell-cell and cell-matrix adhesion that is in agreement with more invasive phenotype of this type of cancers. Whereas the proliferative type is largely enriched with copy number gains mainly distributed in the genes responsible for maintaining cell survival (Figure 1.2).

Evidently, NaviCell Web Service can be used not only for visualization of the data themselves but also for visualizing the results of statistical analysis, such as a set of genes with pre-computed t-test values, characterized by p-values (Figure 1.5). In this way, only statistically significant differences can be presented to the user.

Mesenchymal type: EMT map **Proliferative type: Survival map**

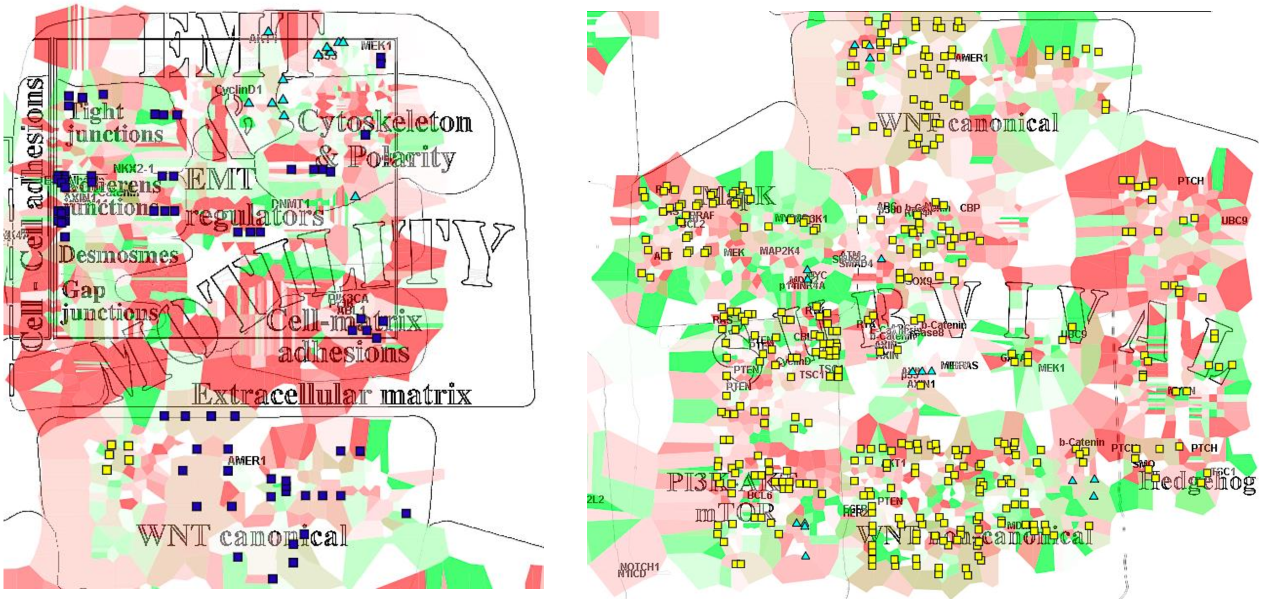

Figure 1.2. Simultaneous visualization of thee type of data for two intrinsic types ovarian cancer, as detailed in Figure 1.1. Zoom in at the individual biological processes differentially regulated at the level of copy number variations in mesenchymal and proliferative types of ovarian cancer.

The most affected by copy number losses are genes from adhesion junctions as cadherins in the mesenchymal type of cancer. The most enriched area with the copy number gains in the proliferative type is Wnt-non canonical pathway, with the key players as FZD1, calpain , PKA (Figure 1.3).

Mesenchymal type: Cell-cell adhesion signaling **Proliferative type: Wnt non-canonical signaling**

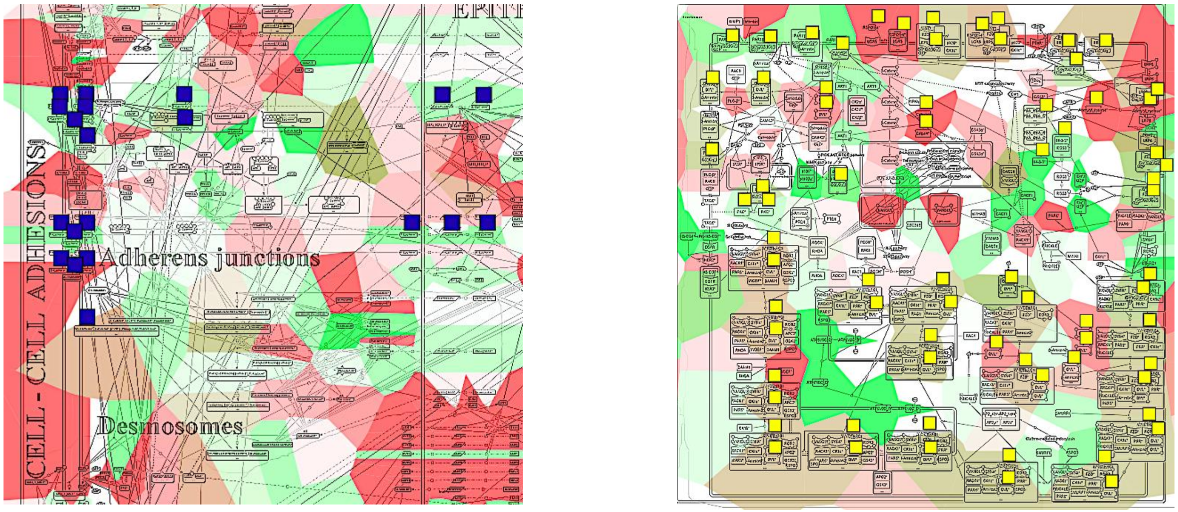

Figure 1.3. Simultaneous visualization of three types of data for two intrinsic types of ovarian cancer from Figure 1.1. Zoom in the signaling pathways enriched with copy number variations in mesenchymal and proliferative types of ovarian cancer.

It is well established that kinases are often mutated in cancers, in majority of cases the most affected are common kinases, but in some cases the mutations are cancer-specific.

In our example, the mutations are also non-equally distributed across the four types of ovarian cancer. The mesenchymal type of ovarian cancer is especially enriched with mutations in various members of Receptor Tyrosine Kinase (RTK) family. On contrary, the proliferative type of ovarian cancer contains mutations in Leucine-Rich Repeat Kinase 2 (LRRK2), the kinase that rarely involved in cancers.

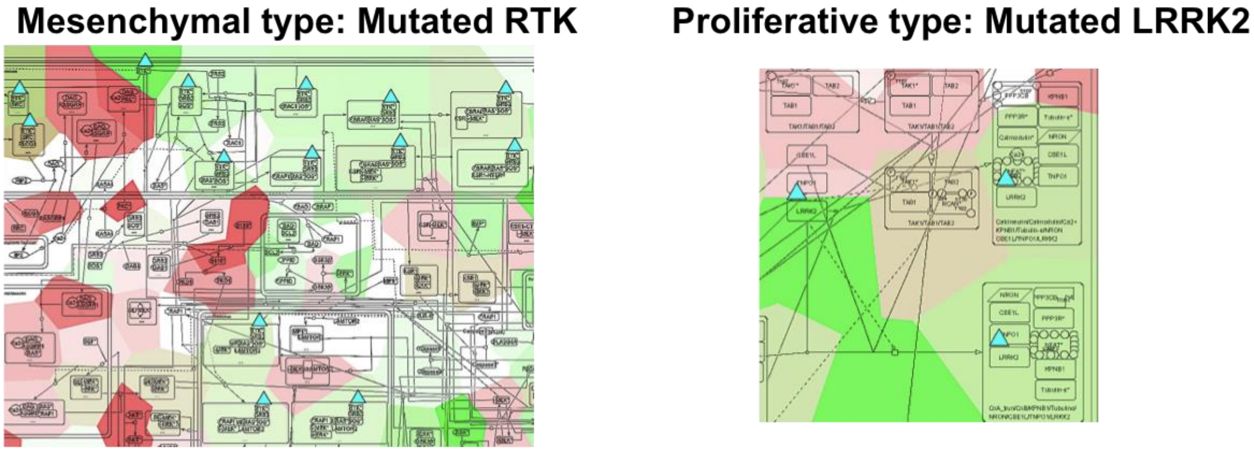

Figure 4. Simultaneous visualization of three types of data for two intrinsic types of ovarian cancer from Figure 1.1. Zoom in some of the mutated genes in mesenchymal and proliferative types of ovarian cancer.

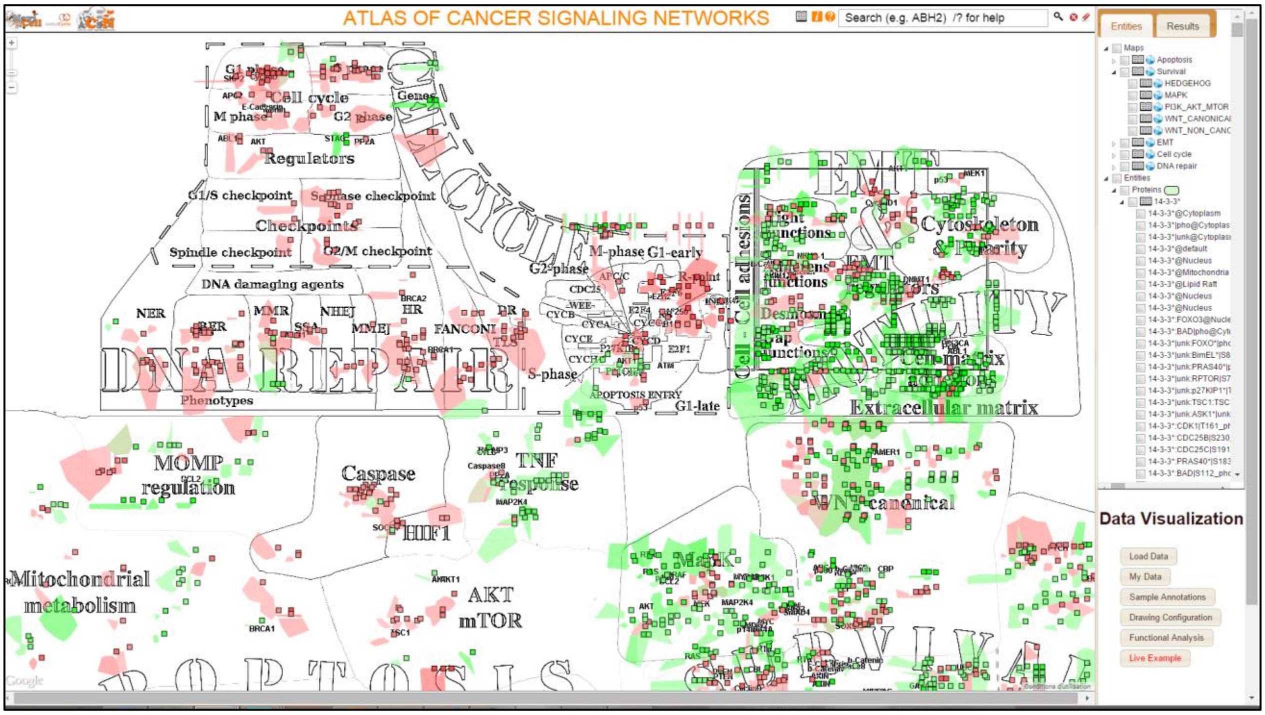

Figure 1.5. Visualization of the results of statistical analysis for comparing two types of ovarian cancer (Proliferative vs Mesenchymal, see also Figure 1.1). The values of the computed t-tests have been used for visualization. The threshold is chosen to show genes with p-value<0.01. Red color illuminates the genes upregulated in Proliferative type, green – the genes which are upregulated in Mesenchymal subtype.

References:

1. The-Cancer-Genome-Atlas-Research-Network (2011) Integrated genomic analyses of ovarian carcinoma. Nature, 474(7353), 609–615.
2. Kuperstein I, Cohen DP, Pook S, Viara E, Calzone L, Barillot E, Zinovyev A. NaviCell: a web-based environment for navigation, curation and maintenance of large molecular interaction maps. 2013.*BMC Syst Biol* **7**(1):100.

CASE STUDY 2 : Using NaviCell Web Service for visualization of transcriptome dynamics after shRNA-based inhibition of EWS/FLI-1 chimeric oncogene in Ewing's sarcoma inducible cell line

This case study demonstrates the applicability of NaviCell Web Service for data visualization on different type of molecular interaction maps (e.g. generated not in the CellDesigner tool), as well as using data visualization for hypothesis formulation which can be subject of further statistical analysis

Ewing sarcoma is the second most frequent pediatric bone tumor. In most of the patients, a chromosomal translocation leads to the expression of the EWS-FLI1 chimeric transcription factor that is the major oncogene in this pathology. Relative genetic simplicity of Ewing sarcoma makes its particularly attractive for studying cancer in a systemic manner. Silencing EWS-FLI1 induces cell cycle alteration and ultimately leads to apoptosis. For studying the mechanisms of this phenomenon, a network linking EWS-FLI1 to cell cycle and apoptosis phenotypes was constructed through original format and method of network reconstruction (Stoll et al., 2013). Transcriptome time-series after EWS-FLI1 silencing were used to identify core modulated genes by an original scoring method based on fitting curves.

The network prepared in Cytoscape format (see Supplementary materials for Stoll et al, 2013) was converted into a CellDesigner file using BiNoM Cytoscape plugin (Zinovyev et al, 2008; Bonnet et al, 2013). For visualization in NaviCell (Kuperstein et al, 2013), the original image of the network was used. Dynamical transcriptomic data used in (Stoll et al, 2013) for network reconstruction were visualized using the functions of NaviCell Web Service (see Figure 2.1). The data depict the temporal changes in the expression of all the genes (measured by Affymetrix microarray technology) followed silencing expression of EWS/FLI-1 in cell lines by shRNA. There are in total 10 time points measured during 17 days after EWS/FLI-1 silencing.

Map staining technique allows evaluating the global changes in the transcriptome of the tumoral cells (Figure 2.2). This visualization illustrates the switch of a cancer cell state from tumorigenic and proliferative (DAY0) to apoptotic and non-proliferative (e.g., DAY 9). Note increased expression of the genes regulating cell motility (bottom part of the network) as a result of EWS/FLI-1 inactivation: this is an unexpected effect which can be interesting for biological validation. This effect becomes even more striking when visualizing the same transcriptomes on top of ACSN map (http://acsn.curie.fr) and allows formulating a hypothesis on that less proliferative state of Ewing's sarcoma cells is characterized by activated ability to interact with extracellular matrix and eventually migrate (Figure 2.3A,B). This hypothesis was not expected but it can be supported by rigourous statistical analysis (Figure 2.3C).

This case study illustrates using NaviCell Web Service (https://navicell.curie.fr/pages/nav_web_service.html) for visualization of dynamic data, using the networks which were not initially prepared in CellDesigner format. Using BiNoM functions, NaviCell Web Service can be applied to practically any networks which can be loaded into Cytoscape environment (eg, from a BioPAX file).

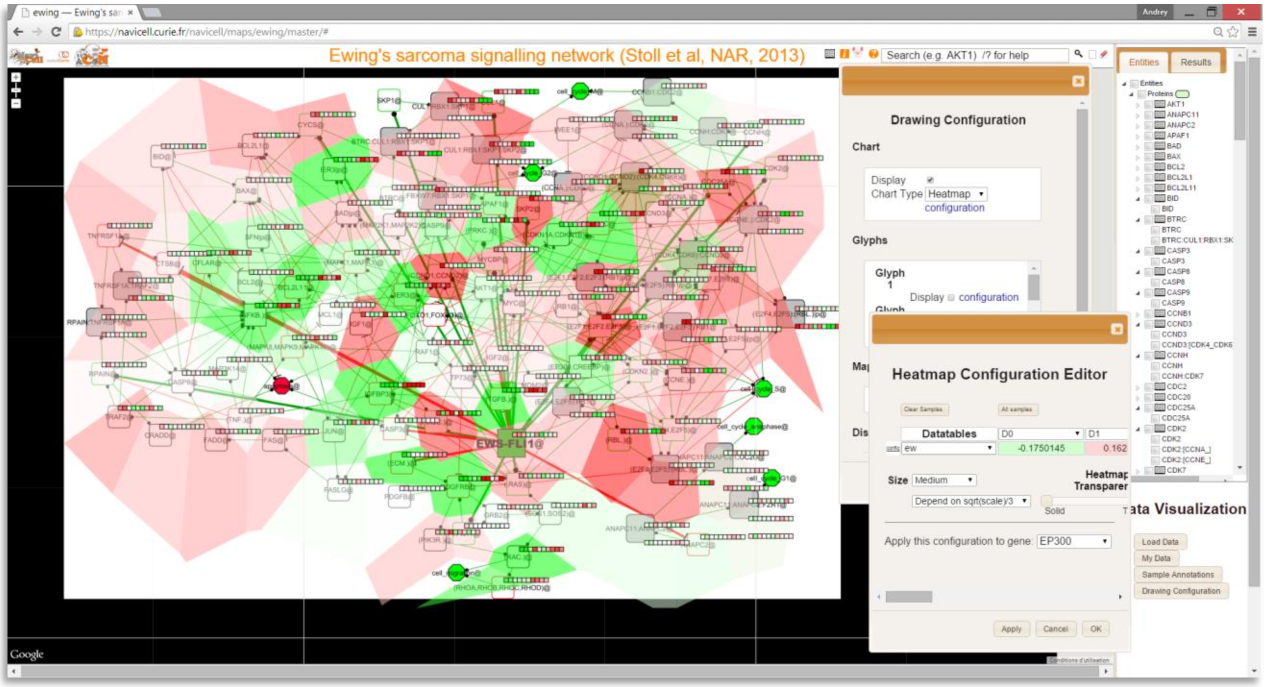

Figure 2.1. Screenshot of NaviCell Web Service GUI with the network of downstream effects of EWS/FLI-1 chimeric oncogene (Stoll et al, 2013). Dynamic transcriptomic data visualized on top of it. Map staining shows expression at day 0, when EWS/FLI-1 is expressed. Heatmap method is used to visualize expression of the genes for the whole time series (10 time points, 17 days after inhibition). The color gradient visualizes the continuous scale of expression values, centered around the average value for each gene. Red color signifies elevated expression, green – decreased expression, and white – close to average values.

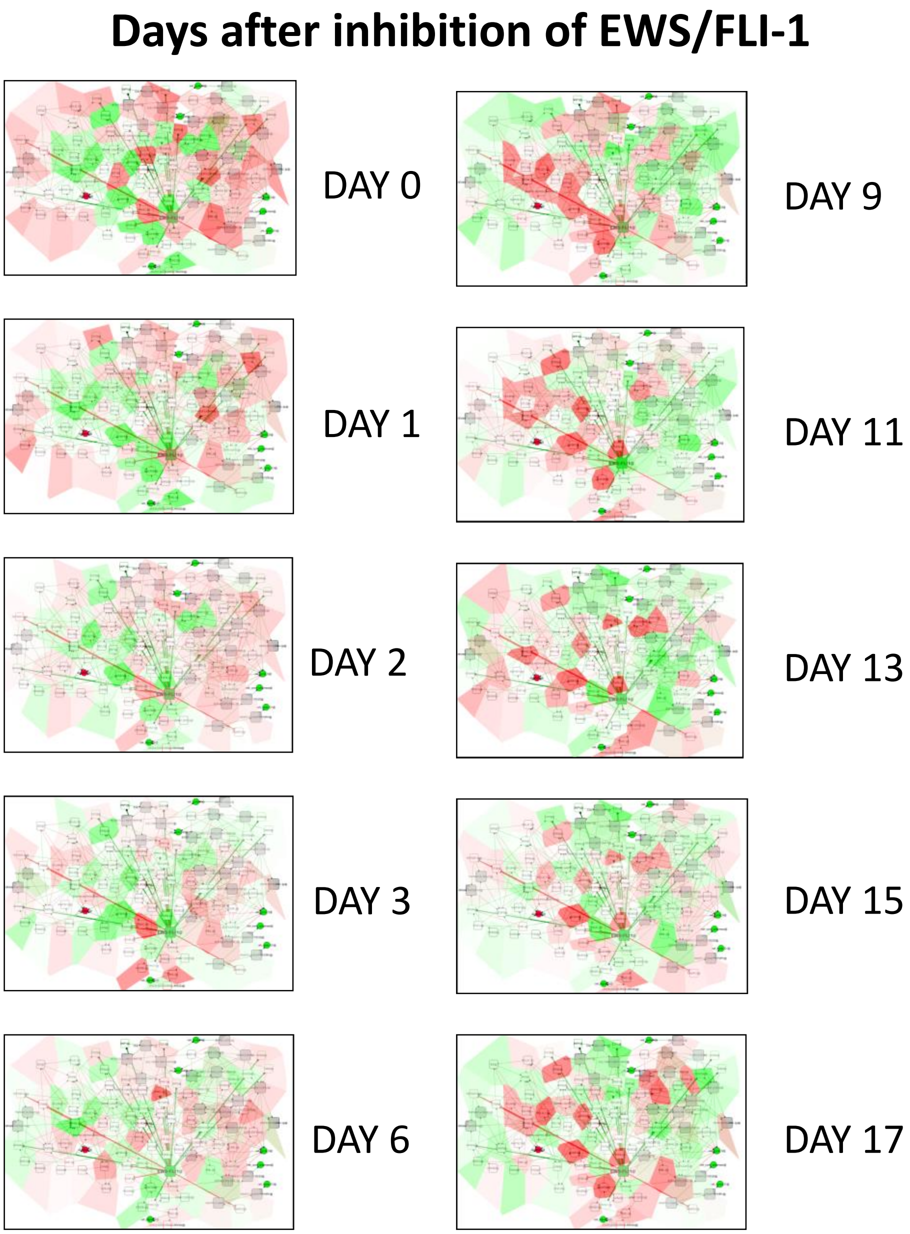

Figure 2.2. Visualization of the transcriptome dynamics using map staining technique.

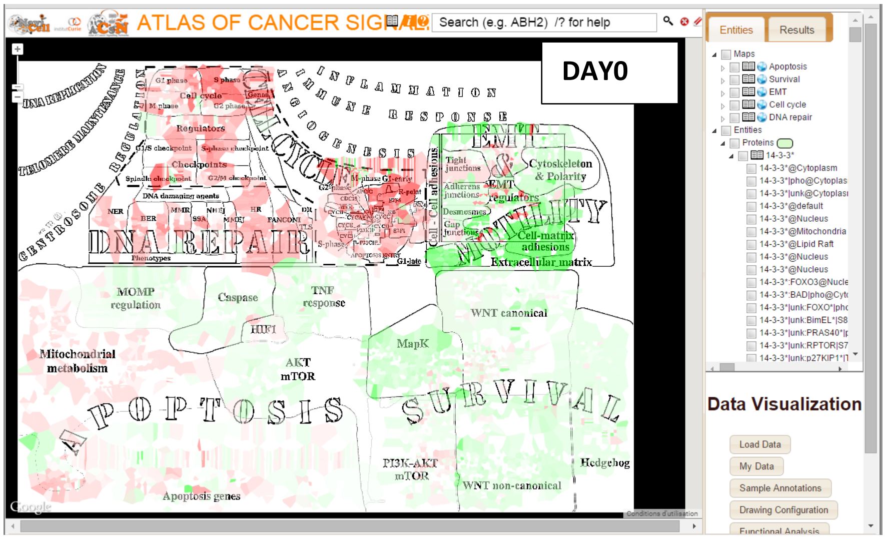

A)

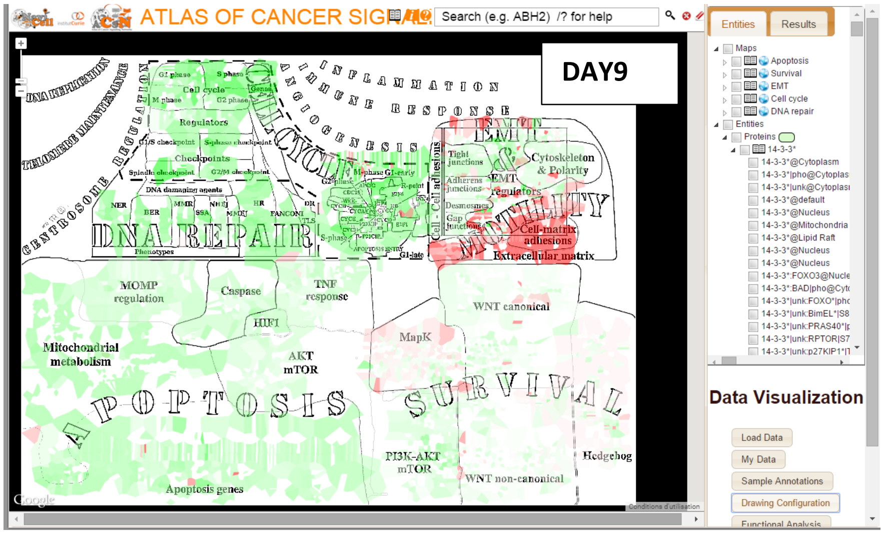

B)

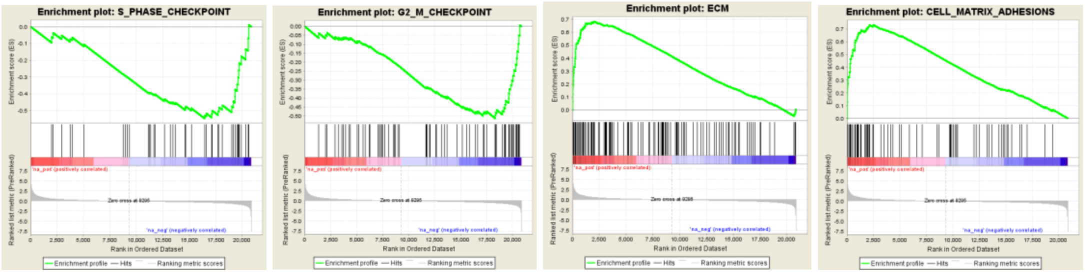

p-value=0.0024 p-value<1e-4 p-value>1e-4 p-value<1e-4 C)

Figure 2.3. Visualization of the transcriptome dynamics using map staining technique on top of Atlas of Cancer Signaling Network (http://acsn.curie.fr). A) Top level ACSN map with visualization of inducible cell line transcriptome at DAY0, before inhibiting the EWS/FLI-1 oncogene; B) same as A) but showing the transcriptome at DAY9 after inhibition of EWS/FLI-1; C) Comparison of A and B suggests a switch between proliferative and migrating states of the Ewing's sarcoma cells (visible as drastic change of the color of Cell Cycle and EMT/Cell Motility maps, and especially Cell-matrix adhesion module) appearing between day 0 and day 9 of the experiment; this hypothesis formulated from data visualization was further supported by rigorous statistical analysis, using GSEA, using difference between expression values in Day 9 and Day 0 as a score for calculating the enrichment score of the map modules.

References:

1. Stoll G, Surdez D, Tirode F, Laud K, Barillot E, Zinovyev A, Delattre O. Systems biology of Ewing sarcoma: a network model of EWS-FLI1 effect on proliferation and apoptosis. 2013. *Nucleic Acids Res.*, **41**(19):8853-71.
2. Bonnet E, Calzone L, Rovera D, Stoll G, Barillot E, Zinovyev A. BiNoM 2.0, a Cytoscape plugin for accessing and analyzing pathways using standard systems biology formats. 2013. *BMC Syst Biol.***7**(1):18.
3. Zinovyev A., Viara E., Calzone L., Barillot E. BiNoM: a Cytoscape plugin for using and analyzing biological networks. 2008. *Bioinformatics* **24**(6):876-877
4. Kuperstein I, Cohen DP, Pook S, Viara E, Calzone L, Barillot E, Zinovyev A. NaviCell: a web-based environment for navigation, curation and maintenance of large molecular interaction maps. 2013.*BMC Syst Biol* **7**(1):100.
